# SnO_2_ Films Deposited by Ultrasonic Spray Pyrolysis: Influence of Al Incorporation on the Properties

**DOI:** 10.3390/molecules24152797

**Published:** 2019-07-31

**Authors:** Getnet Kacha Deyu, David Muñoz-Rojas, Laetitia Rapenne, Jean-Luc Deschanvres, Andreas Klein, Carmen Jiménez, Daniel Bellet

**Affiliations:** 1 Univ. Grenoble Alpes, CNRS, Grenoble INP, LMGP, 38 000 Grenoble, France; 2 Electronic Structure of Materials, Department of Materials and Earth Sciences, Technische Universität Darmstadt, Otto-Berndt-Straße 3, 64287 Darmstadt, Germany

**Keywords:** tin oxide, aluminum, spray pyrolysis, doping, transparent conductive oxide

## Abstract

Aluminum-doped tin oxide (SnO${}_{2}$:Al) thin films were produced by an ultrasonic spray pyrolysis method. The effect of aluminum doping on structural, optical, and electrical properties of tin oxide thin films synthesized at 420 ${}^{{}^{\circ}}$C was investigated. Al doping induced a change in the morphology of tin oxide films and yielded films with smaller grain size. SnO${}_{2}$ thin films undergo a structural reordering and have a texture transition from (301) to (101), and then to (002) preferred cristallographic orientation upon Al doping. The lattice parameters (*a* and *c*) decreases with Al doping, following in a first approximation Vegard’s law. The optical transmission does not change in the visible region with an average transmittance value of 72–81%. Conversely, in the near infrared (NIR) region, the plasmon frequency shifts towards the IR region upon increasing Al concentration in the grown films. Nominally undoped SnO${}_{2}$ have a conductivity of ∼1120 S/cm, which is at least two orders of magnitude larger than what is reported in literature. This higher conductivity is attributed to the Cl${}^{-}$ ions in the SnCl${}_{4}$·5(H${}_{2}$O) precursor, which would act as donor dopants. The introduction of Al into the SnO${}_{2}$ lattice showed a decrease of the electrical conductivity of SnO${}_{2}$ due to compensating hole generation. These findings will be useful for further studied tackling the tailoring of the properties of highly demanded fluorine doped tin oxide (FTO) films.

## 1. Introduction

Research in tin oxide (SnO${}_{2}$) is gaining dramatic interest among the wide-band gap semiconductor community due to its unique photoelectric and outstanding electrical conduction properties. Furthermore, doping of SnO${}_{2}$ with metal ions has been used to tailor the properties of the base material, which in turn has resulted in an enhancement of the device performance [1,2,3,4]. This makes SnO${}_{2}$ thin films excellent candidates for large-scale applications in gas sensors [5,6], solar cells [7,8], lithium-ion batteries [9,10], low emission window [1], and UV photodetectors [11].

The material is n-type by nature due to an intrinsic doping mechanism [12] with a band gap of 3.6 eV and can be highly n-doped by addition of extrinsic dopant elements such as F, Sb, Ta, or Nb; p-doped by Li, or Cd doping; and isovalent by Ti doping among others [13,14,15].

To comply with the specification imposed by the numerous applications, different SnO${}_{2}$ properties are required. Undoped and surface doped SnO${}_{2}$ thin films are very good candidates for semiconductor based gas sensing applications (Taguchi sensors) due to a naturally occurring high oxygen non-stoichiometry accommodated by the SnO${}_{2}$ lattice [5]. For this application, a variation of the electrical conductivity of a material as a function of the atmosphere is required. A well accepted model to explain such change of conductivity is based on the modification of band bending in the near-surface induced by adsorption/desorption of gas molecules on/from the oxide surface [16,17]. Doped SnO${}_{2}$ thin films are also used as front electrodes in optoelectronic devices such as thin film solar cells, displays and light emitting diodes [18]. In this field of application, tin oxide is an alternative to other transparent conductive oxide (TCO) materials such as Sn-doped In${}_{2}$O${}_{3}$ (ITO) [19] and Al-doped ZnO [20]. Although higher conductivity have been achieved with the two latter oxides, SnO${}_{2}$ based TCO materials offer enhanced chemical, mechanical, and thermal stability, as well as the relative abundance of tin ores in the earth’s crust [1]. Another wide application area of SnO${}_{2}$ is in low-emission window coatings, due to its high stability in various environmental conditions. The possibility to induce a high carrier concentration, typically by F-doping, results in the shifting of the plasma frequency in the near IR-region, thus resulting in an enhanced reflection of low energy heat radiation.

A wide variety of growth methods can typically be used for the deposition of undoped and differently doped tin oxide thin films, including magnetron sputtering [20,21,22,23], molecular beam epitaxy [24], pulsed-laser deposition (PLD) [25], chemical vapor deposition (CVD) [26], atomic layer deposition (ALD) [27], or spray pyrolysis [28,29,30]. Spray pyrolysis has the advantages of being cost efficient and surface scalable [31].

To assist the design of SnO${}_{2}$-based nanomaterials for widespread use, it would be helpful to understand the role of dopants in the SnO${}_{2}$ thin films. Thus far, a limited number of studies have been reported on Al-doped SnO${}_{2}$ thin films deposited by various deposition methods including PLD [32], spray pyrolysis [33,34], sol-gel [35], powder sintering [36], and ALD [27]. However, detailed studies on the effects of Al doping on the structural, optical, and electrical properties of tin oxide films are still lacking. In this contribution, aluminum (Al) doped SnO${}_{2}$ thin films prepared by ultrasonic spray pyrolysis are investigated. The influence of Al incorporation with different concentration in tin oxide thin films on structural, optical, and electrical properties is examined.

## 2. Materials and Methods

Polycrystalline nominally undoped and Al-doped SnO${}_{2}$ thin films were prepared by a homemade ultrasonic spray pyrolysis deposition system [28,30,37] on different substrates, namely Si wafer, Corning C1737 borosilicate glass, fused silica glasses, and SiO${}_{2}$/Si wafer. The tin precursor was SnCl${}_{4}$·5(H${}_{2}$O) dissolved in methanol with a fixed concentration of 0.1 M. To incorporate aluminum in the SnO${}_{2}$ thin films, aluminum acetylacetonate, Al(acac)${}_{3}$ was added in different concentrations: 0.0005, 0.001, 0.0015, 0.002, 0.0025, 0.005, 0.01, and 0.015 M; corresponding to the Al/(Al+Sn) atomic ratio in a solution of 0%, 0.5%, 1%, 1.37%, 1.96%, 2.44%, 4.76%, 9.1%, and 13.04%. During deposition of the samples, the growth temperature was set at 500 ${}^{{}^{\circ}}$C, which resulted in a substrate surface temperature of 420 ${}^{{}^{\circ}}$C. This temperature setting was kept constant for all the substrates and depositions.

A detailed characterization study was conducted using a wide range of standard analysis techniques. The surface morphology of different samples was analyzed by field emission gun-scanning electron microscopy (FEG-SEM Environmental FEI QUANTA 250). Top-view images were processed using digital ImageJ software in order to determine both the average and the biggest grain sizes in each film. The aluminum content in the prepared samples was estimated using an energy dispersive spectroscopy (EDS) analyzer equipped with a FEG-SEM system and Electron Probe Micro-Analyzer (EPMA, Cameca SX50 system equipped with wavelength dispersive spectrometers). EDS analyses were performed at low acceleration voltage (6 keV) to limit the signal obtained from the substrate so that a low atomic number element in a low concentration could be detected. EMPA measurements were performed at three different electron beam acceleration voltage values (11, 16, and 22 keV) and the data were analyzed using the Stratagem program dedicated to the analysis of thin films [38]. Transmission electron microscopy (TEM) imaging was performed with a JEOL JEM 2010 microscope operating at 200 kV (0.19 nm resolution), equipped with energy-dispersive X-ray spectroscopy (EDX). Bragg–Brentano (θ-2θ) X-ray diffraction (XRD) patterns were collected with a Bruker D8 Advance Series II diffractometer (Billerica, MA, USA) using CuK$\alpha_{1}$ radiation in the 2θ range of 10–70${}^{{}^{\circ}}$. Fourier-transform infrared (FTIR) measurements were performed in a Brucker Vertex 70 V spectrometer equipped with CsI beamsplitter and working under vacuum. Spectra were recorded with a resolution of 4 cm${}^{-1}$ by accumulating 64 scans in transmission mode. A UV-Vis-NIR spectrophotometer equipped with an integrating sphere (PerkinElmer Lambda 950, Waltham, MA, USA) was used to record the optical transmittance between 250 and 2500 nm. Hall effect measurements were performed at room temperature in the classical Van der Paw configuration using a homemade setup operating under a magnetic field of 0.5 T.

## 3. Results and Discussion

**Figure 1:**
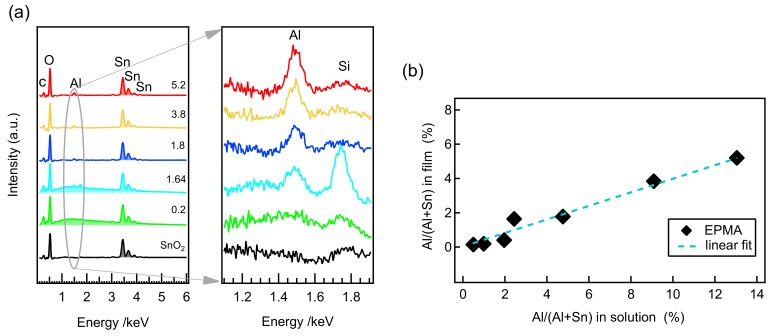
(**a**) EDS spectra at 6 keV of different SnO${}_{2}$:Al thin films prepared on Si wafer substrates. In the right, magnified spectra of the same films around 1.5 keV is shown to see the evolution of Al peak; (**b**) relative Al content as obtained from EPMA compared to the amount in the precursor solution. A dashed line represents the linear relationship.

In order to evaluate the incorporation of aluminum in the SnO${}_{2}$ thin films, EDS and EPMA measurements were performed on the films deposited on Si wafer substrates; the results are presented in Figure 1. EDS confirmed the presence of Al in the deposited tin oxide thin films. The aluminum peak at 1.486 keV intensifies as the amount of aluminum in the precursor solution increased, as shown in the magnified image of Figure 1a. The aluminum incorporation in each individual film was quantified from the EPMA analyses.

The Al/(Al+Sn) atomic ratio obtained from EPMA is presented in Table 1 and the same results are plotted versus Al/(Al+Sn) atomic ratio in the starting solution as shown in Figure 1b. The aluminum content in the produced films is only about ∼1/3 of that in the precursor solution. This suggests that, under the current selected deposition conditions, tin oxide is more efficiently deposited than aluminum is incorporated.

For the sake of simplicity for presenting the results and further interpretation and discussion, sample identification is given here by using Al/(Al+Sn) results from EPMA, summarized in Table 1. Undoped tin oxide will be named as nominally undoped SnO${}_{2}$ or Al doped tin oxide films (SnO${}_{2}$:Al) are differentiated as SnO${}_{2}$:Al-XX, with XX corresponding to the Al concentration.

**Figure 2:**
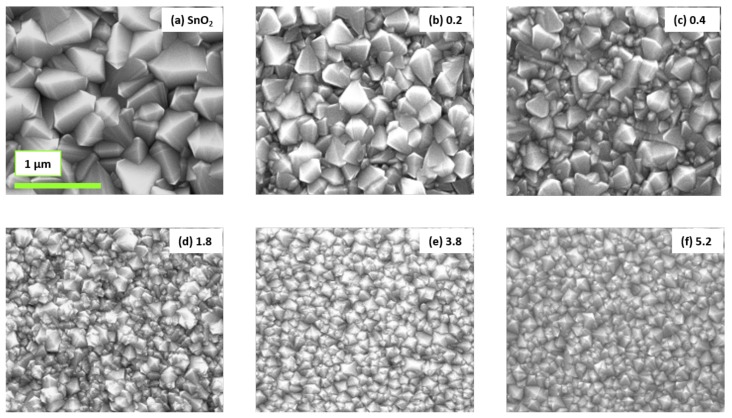
Top-view FEG-SEM images of nominally undoped SnO${}_{2}$ and different Al-doped SnO${}_{2}$ thin films deposited on silicon wafer. (**a**) represent the pure SnO${}_{2}$, while (**b**)–(**f**) corresponding to Al-doped films with the number associated with each image is the measured atomic ratio (Al/Al+Sn) in the films. All images have the same scale of 1 μm and the green scale bar presented in image (**a**) will be used for all images.

The top view SEM images of nominally undoped SnO${}_{2}$ and the different Al-doped SnO${}_{2}$ films prepared on Si wafer substrates are shown in Figure 2. The thicknesses of these films were estimated from SEM cross-section measurements and are displayed in Table 1. The thickness variations among these films are mainly due to different deposition times (25, 30, and 45 min) and a slight variation of average flow rate of the precursor solution (2–2.3 mL/min). The corresponding deposition rates are also given in Table 1. The produced films are all crystalline and significant morphological changes are observed upon Al incorporation. A similar trend has been observed for the films prepared on the other substrates.

The SEM image of nominally undoped SnO${}_{2}$ film clearly reveals the polycrystalline nature of the films as well as the presence of extended planar twin defects crossing the entire grains. Moreover, the density of these defects is high as several extended twin defects can be identified within individual grains. Similar observations have been reported by different researchers for SnO${}_{2}$ films produced by spray pyrolysis and the density of twins increased with increasing the concencentration of tin precursor SnCl${}_{4}$·5(H${}_{2}$O) [28,29,39], along with an increase of deposition rate. It is important to note that these twin boundaries are not desirable since they are considered as an additional electron scattering sites, thus resulting as being detrimental for the electrical properties of SnO${}_{2}$ [37]. Although the density of planar defects decreased considerably upon doping, they can also be seen in the SnO${}_{2}$:Al-0.2 and 0.4 films; see (b) and (c) images of Figure 2, while, for higher Al content films (1.8–5.2), the lamellar twins are not present anymore.

**Figure 3:**
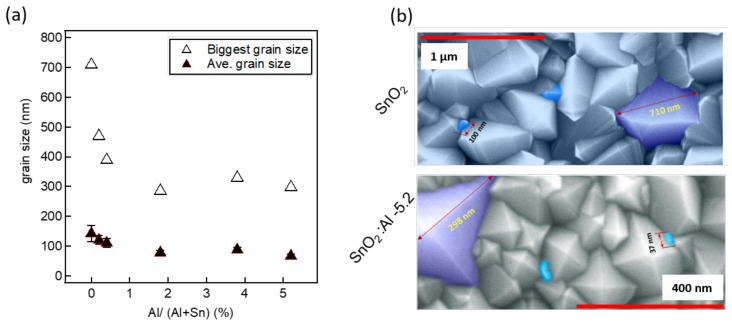
(**a**) the change of average grain size in tin oxide thin films versus Al cationic ratio in the film: the biggest grain within the same films are also represented; (**b**) visual illustration of the size dispersion in the studied samples: SEM images of nominally undoped SnO${}_{2}$ and SnO${}_{2}$:Al-5.2 films are shown. In both cases, the biggest grains are colored in violet, while some smaller grains are colored in sky blue for comparison.

Due to the presence of both grains and twin boundaries in nominally undoped SnO${}_{2}$ and some of the Al-doped films, it is important to distinguish between the grain size (L${}_{g}$) and crystallite size (L${}_{c}$) of the studied films. The grain size (L${}_{g}$) of the studied films was determined from top view SEM images shown in Figure 2, by using the digital image processing ImageJ software. The average and the biggest grain size of these films are plotted for different compositions of SnO${}_{2}$:Al thin films—see Figure 3a. The visual observation of SEM images of Figure 2 and the grain size plot of Figure 3a shows that the grain size decreased consistently with increasing alumina concentration in the precursor solution. This could result from a thermodynamically favored hetrogeneous nucleation of small grains induced by Al incorporation. Similar observations have been reported by Sinha et al. [32], Ahmed et al. [35], and Moharrami et al. [40]. Nominally undoped SnO${}_{2}$ have an average grain size of 142 ± 27 nm with the biggest grain of the same film having a size of 710 nm, which indicates that the grain size range can span up to a factor of 6 within the same film. Similarly, for the highest Al content SnO${}_{2}$:Al-5.2 film, the average grain size is 67 ± 6 nm with the biggest grain being 300 nm, which also shows five times grain size variations within the film. These results are supported by the SEM images of the same films shown in the top and bottom images of Figure 3b. Figure 3a indicates that the Al composition within SnO${}_{2}$:Al films plays a key role in the final grain size, more than the film thickness for instance. Indeed, the dependency of the average or biggest grain size exhibit a monotonous dependency and is rather independent from film thickness, at least in the range of 200–800 nm, see Table 1. The physical origin of these observations is probably a favored heterogeneous nucleation induced by Al incorporation in the film.

**Figure 4:**
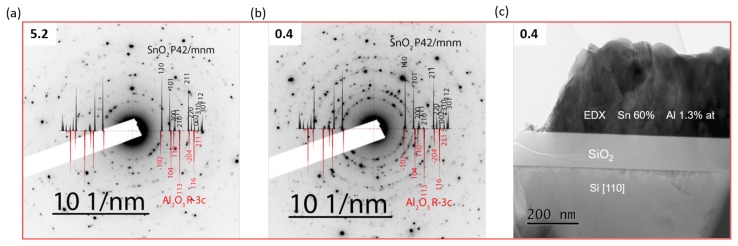
TEM electron diffraction patterns of different as deposited and post annealed SnO${}_{2}$:Al films. (**a**) diffraction patterns of as deposited SnO${}_{2}$:Al-5.2 film prepared on Si wafer substrate with indices of SnO${}_{2}$ (ICDD-00-041-1445) and α-Al${}_{2}$O${}_{3}$ (ICDD-00-046-1212); (**b**) diffraction pattern of SnO${}_{2}$:Al-0.4 film prepared on SiO${}_{2}$/Si substrate, which was further post annealed at 1000 ${}^{{}^{\circ}}$C for one hour in air, with indices of both SnO${}_{2}$ and α-Al${}_{2}$O${}_{3}$; and (**c**) EDX of the same SnO${}_{2}$:Al-0.4 sample which reveals the presence of Al 2.1% as cationic content.

TEM samples were prepared by scratching the surface using a diamond tip to scratch off particles on holey carbon copper grids. TEM analyses were performed to assess whether Al was completely incorporated into the tin oxide lattice structure or rather was incorporated as a secondary crystalline Al${}_{2}$O${}_{3}$ phase. For this purpose, two different compositions of SnO${}_{2}$:Al-0.4 and -5.2 were selected and TEM electron diffraction and EDX analyses were performed, as shown in Figure 4. Al${}_{2}$O${}_{3}$ can exist in different crystalline phases depending on its purity and different growth conditions. The polymorphs of alumina include γ,δ,κ, θ, and α, from which the α-phase is thermodynamically stable and is obtained at very high temperature ≥1000 ${}^{{}^{\circ}}$C [41].

SnO${}_{2}$:Al-5.2 is the sample with the highest Al content in the studied composition series. The sample deposited on Si was studied by TEM analysis without further post annealing treatment. As can be seen in Figure 4a, the obtained diffraction rings are in good agreement with the indices of SnO${}_{2}$. As for Al${}_{2}$O${}_{3}$, there was no single diffraction ring matching the indices of crystalline alpha phase or any other alumina polymorphs.

Since as deposited SnO${}_{2}$:Al-5.2 film does not show any crystalline Al${}_{2}$O${}_{3}$ phase, another composition of SnO${}_{2}$:Al-0.4 film deposited on SiO${}_{2}$/Si substrate was selected and further post annealed at 1000 ${}^{{}^{\circ}}$C for one hour in air before TEM analysis. This composition was selected due to its better electrical properties than that of SnO${}_{2}$:Al-5.2. Both the diffraction pattern and EDX results are presented in Figure 4b,c.

Similar to the as-deposited SnO${}_{2}$:Al-5.2 sample, the diffraction rings of this film are also in good agreement with indices of SnO${}_{2}$ and there was no diffraction ring matching the indices of α-Al${}_{2}$O${}_{3}$ or other alumina polymorphs. Thus, even though the sample was post annealed at 1000 ${}^{{}^{\circ}}$C, the TEM diffraction does not show a contribution associated with any polymorph of crystalline Al${}_{2}$O${}_{3}$. Actually, at this annealing temperature, theta or alpha phases would be present [41,42], which we do not see in our case. The possible reason for the observed absence of the diffraction rings of Al${}_{2}$O${}_{3}$ phases could be that the diffraction rings of SnO${}_{2}$ and Al${}_{2}$O${}_{3}$ are very close to each other. For example, the interreticular plane distance of SnO${}_{2}$ (110) is 3.349 Å and that of Al${}_{2}$O${}_{3}$ (012) 3.48 Å; this is also true for other peaks. In addition, Al${}_{2}$O${}_{3}$ is in a very low quantity phase in the studied compositions. Hence, it is possible to have some spots of Al${}_{2}$O${}_{3}$ in the diffraction images but also it is difficult to identify them. The EDX analysis of the same sample revealed the presence of a 2.1% of Al as cationic content. Therefore, it is reasonable to conclude that if not all, most of Al was incorporated into tin oxide lattice for the studied films.

**Figure 5:**
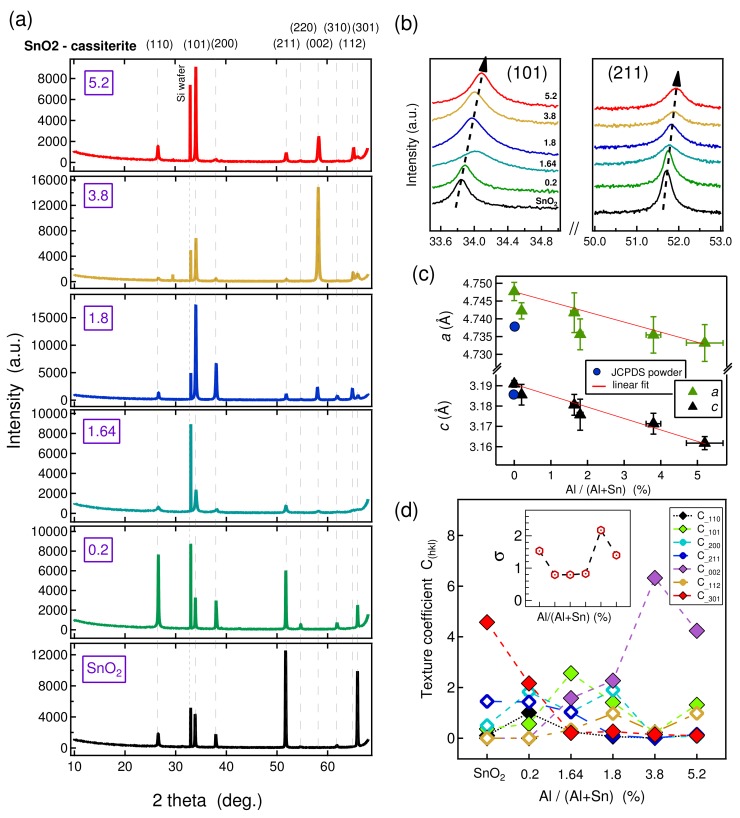
(**a**) X-ray diffraction (XRD) θ-2θ patterns of nominally undoped SnO${}_{2}$ and different Al-doped SnO${}_{2}$ thin films with increasing Al content in the prepared samples using Si wafer substrate. In the same plot, the diffraction peaks corresponding to SnO${}_{2}$ are marked with dashed lines (PDF-00-041-1445) and labeled with individual crystallographic orientation on the top; (**b**) demonstration for broadening and shifting to higher angles of XRD patterns of tin oxide films upon Al incorporation. Here, two dashed black arrows are used to indicate that the Bragg peak position is continuously increased with increasing Al content in the grown films; (**c**) lattice constants *a* and *c* vs. Al cationic (%) in the film. The lattice constants of standard SnO${}_{2}$ JCPDS powder are also included for comparison. The linear interpolations (red lines) between SnO${}_{2}$ and SnO${}_{2}$:Al-5.2 films are plotted to show the linear relationship between lattice constant and Al concentration in the films following Vegard’s law; (**d**) the change of texture coefficients C${}_{hkl}$ calculated for tin oxide and different Al-doped SnO${}_{2}$ films plotted for different SnO${}_{2}$:Al compositions. The color code of different crystallographic orientations (hkl) which are used to calculate C${}_{hkl}$ are represented in the legend. The plot for the degree of preferred orientation σ of the same films also shown in the insert. Note that, for a better visual, the *x*-axis of (**d**) is not in a linear scale.

The θ-2θ XRD diffraction patterns of undoped SnO${}_{2}$ and different Al-doped SnO${}_{2}$ thin films collected between 10${}^{{}^{\circ}}$ and 70${}^{{}^{\circ}}$ (in 2θ scale) are shown in Figure 5a. The XRD spectra match with high tetragonal SnO${}_{2}$ with PDF card (00-041-1445), with no Al${}_{2}$O${}_{3}$ polymorph being detected. This is another indication that Al was successfully incorporated into the tin oxide films without forming secondary crystalline Al${}_{2}$O${}_{3}$ phases.

The XRD patterns of probed samples exhibit a systematic shift to higher degree and a broadening of the peaks upon increasing Al content in tin oxide films. To show this phenomenon, the (101) and (211) diffraction peaks were selected and plotted for all samples, in Figure 5b. For the (101) reflection, the peak position shifted from 33.84${}^{{}^{\circ}}$ for nominally undoped tin oxide to 34.089${}^{{}^{\circ}}$ for the SnO${}_{2}$-5.2 sample. Similarly, the (211) reflection exhibited a shift of 0.23${}^{{}^{\circ}}$ to a higher angle for the SnO${}_{2}$-5.2 sample compared to nominally undoped SnO${}_{2}$. In both reflections, black dashed arrows were used for visual guidance of the peaks shifting to higher angle. In addition, broadening of the peaks can clearly be observed in both reflections as the Al content increased in the prepared samples.

This shifting of the XRD peaks of tin oxide films upon increasing Al(acac)${}_{3}$ concentration in the precursor solution is attributed to incorporation of Al into the SnO${}_{2}$ lattice structure. In addition, the broadening of the peaks is related to the decrease of the grain size observed upon increasing the Al content. In six-fold coordination, the ionic radii of Al${}^{3+}$ (0.051 nm) is about 30% smaller than that of Sn${}^{4+}$ (0.071 nm), therefore it is compatible with the hypothesis that the Al dopant effectively substitutes the host atom effectively without forming any secondary phase in the system. The overall lattice parameter is thus expected to decrease as more Al ions occupy the Sn sites, as indeed observed experimentally, see Figure 5b. Similar observations have been reported by Lee et al. [27] for Al content in the film (atomic %) varing from 0 to 8.2%, Ravichandran et al. [34] with Al concentration in the starting solution increasing from 0–30 atomic %, and Thirumurugan et al. [43] for 3 atomic % doping. In addition, the broadening of the XRD peaks indicates a diminution of crystal sizes.

The variation of lattice constants (*a* and *c*) of tin oxide films versus Al atomic (%) in the film is presented in Figure 5c. The lattice parameters’ values *a* and *c* were determined from the Equation (1) using (110), (101), (200), and (211) diffraction lines:

(1) $$\frac{1}{d_{hkl}}=\sqrt{\frac{h^{2}+k^{2}}{a^{2}}+\frac{l^{2}}{c^{2}}},$$

where $d_{hkl}$ is the lattice parameter and h,k, and l are the Miller indices.

The errors both in determining the lattice parameters from different diffraction lines and the Al atomic (%) from EPMA measurements are included in the plot. Without doping, the lattice constants of SnO${}_{2}$ are: *a* = 4.74769 ± 0.0026 Å and *c* = 3.19093 ± 0.00164 Å, which are close to the standard SnO${}_{2}$ powder (*a* = 4.73820 Å and *c* = 3.18710 Å). As the dopant concentration increases, both lattice constants decrease considerably and the relative decrease in *c* is almost double that for *a*. To confirm whether the evolution of the lattice parameters upon doping follows Vegard’s law [44,45], which predicts a linear decrease of alloy lattice as the concentration of smaller size dopant increases, a linear fit has been plotted in Figure 5c. With a consideration of both the error bars of lattice parameters and Al atomic % of EPMA measurements, *a* and *c* follow Vegard’s law rather well.

It has been stated that the electrical and optical properties of SnO${}_{2}$ thin films depend on their preferential crystallographic orientations (i.e., texture) [4]. Thus, a deeper understanding and control of the structural ordering is important to adjust these properties. The film texture strongly influences the grain boundary nature, which can affect the electrical mobility by scattering the free charge carriers. Additionally, the film texture governs the crystallographic orientation of the top facets and interface properties in heterojunctions. Interestingly, the preferred orientations are in turn governed by different physical mechanisms and this includes the following parameters by growth conditions, film thickness, type of substrate, nature of chemical precursors, and doping [28,46,47,48].

As dopant ions substitute the host ions, the film growth orientation is altered as a result of mechanical strain induced in the lattice [49,50,51]. Here, the XRD patterns also show significant preferential orientations with Al incorporation into the tin oxide films. The change of texture coefficient C${}_{hkl}$ for SnO${}_{2}$ films upon different percentage of Al doping has been calculated based on θ-2θ XRD measurements of Figure 5a. Only seven main peaks were taken into account: (110), (101), (200), (211), (002), (112), and (301). The texture analysis was quantitatively carried out from the K$\alpha_{1}$ component of each diffraction peak in the framework of the Harris method [52]. The texture coefficients C${}_{hkl}$ for each (hkl) crystallographic directions and degree of preferred orientation σ were respectively defined as the following in Equation (2):

(2) $$C_{hkl}=\frac{\frac{I_{hkl}}{I_{o,hkl}}}{\frac{1}{N}\sum_{N}\frac{I_{hkl}}{I_{o,hkl}}}\;and\;\sigma =\frac{\sqrt{\sum_{N}{\left(C_{hkl}-1\right)}^{2}}}{\sqrt{N}},$$

where $I_{hkl}$ is the intensity of (hkl) reflection of studied samples; $I_{o,hkl}$ is the intensity of the corresponding plane in the reference of powder (PDF 00-041-1445) from the International Center for Diffraction Data (ICDD), and *N* represents the number of reflections, in our case N = 7.

Basically, for randomly oriented samples, the texture coefficient and degree of preferred orientation equal 1 and 0, respectively. In contrast, for perfectly oriented grain samples along the (hkl) direction, the texture coefficient equals N for (hkl) planes (N = 7 in our case) and 0 for other crystallographic planes and consequently the degree of preferred orientation is $\sqrt{N-1}$.

The pronounced structural reordering correlated with the evolution of texture coefficients for each (hkl) crystallographic direction and the degree of preferred orientation of the studied films are shown in Figure 5d. For nominally undoped SnO${}_{2}$, the (301) crystallographic orientation is dominant with a texture coefficient of 4.57 and the (211) crystallographic orientation also has a C${}_{hkl}$ of 1.45. In the case of SnO${}_{2}$:Al-0.2 sample, (301) is still the dominant orientation. In contrast, a texture change is observed for the SnO${}_{2}$:Al-1.64 film. Here, the (101) crystallographic orientation became the dominant orientation with a C${}_{hkl}$ of 2.56. For the SnO${}_{2}$:Al-1.8 sample, the texture changed again and (002) becomes the dominant orientation with a C${}_{hkl}$ of 2.27. Finally, it became the only dominant orientation for SnO${}_{2}$:Al-3.8 and -5.2 films. As a result, polycrstalline SnO${}_{2}$ thin films undergo a texture transition from (301) to (101), and then (002) preferred cristallographic orientation upon increasing Al content in the grown films [53].

It is further shown that the relative intensity between different (hkl) Bragg reflections is strongly dependent on the amount of Al incorporated into the growing film. The degree of preferred orientation is drastically reduced from 1.53 in undoped SnO${}_{2}$ to ≈0.88 for SnO${}_{2}$:Al-0.2 film. It remained almost unchanged between 0.2 and 1.8 films, and then suddenly increased to 2.2 for the SnO${}_{2}$:Al-3.8 film. Finally, for the highest Al content SnO${}_{2}$:Al-5.2 film, the degree of preferred orientation reduced to 1.4 as can be seen in the insert of Figure 5d.

**Figure 6:**
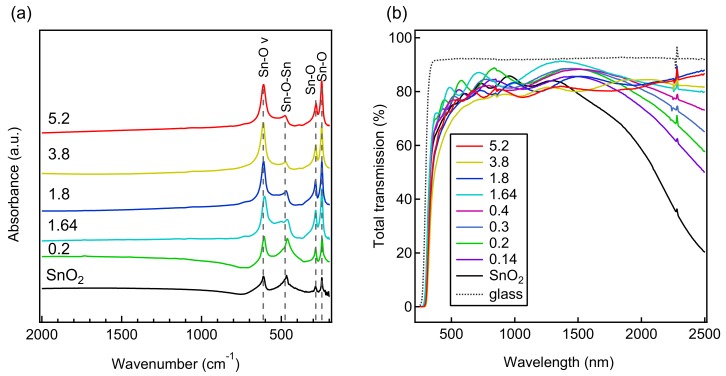
(**a**) normalized FTIR spectra of nominally undoped and different Al-doped tin oxide thin films, (**b**) optical transmittance of nominally undoped SnO${}_{2}$ and different Al-doped SnO${}_{2}$ thin films as a function of wavelength.The transmittance of bare glass is included for comparison.

Films deposited on silicon wafer substrates were further examined by FTIR and the results are presented in Figure 6a. For better visual guidance, all the spectra were normalized with the same intensity for the Si-O v band at 612.5 cm${}^{-1}$. The spectra peaks present at 245, 282.5, and 612.5 cm${}^{-1}$ are assigned to the Sn-O vibration [35,54], while a peak at 468.92 cm${}^{-1}$ is assigned to Sn-O-Sn stretching. Even though there was no independent peak observed which could be assigned to aluminum, Al incorporation has an influence on the broadening of Sn-O-Sn peaks and leads to a decrease of the relative intensity of the Sn-O-Sn stretching mode. This could be attributed to the formation of Sn-O-Al bonds. Actually, Kumar and coworkers [55] reported similar broadening and attenuation of the Sn-O-Sn band as a function of increasing Al content in the SnO${}_{2}$ thin films. Nevertheless, the exact position of Sn-O-Al band is not yet reported in literature or on FTIR handbooks to our knowledge.

The total transmittance spectra of nominally undoped and Al-doped SnO${}_{2}$ thin films on Corning glass substrates measured between 250 and 2500 nm range are shown in Figure 6b. Virtually no shifting of the leading edge at short wavelengths, representing the onset of the leading optical band-to-band absorption (absorption edge), is observed upon Al doping. Thus, there was no blue-shift of the absorption edge. The average total transmittance in the visible (VIS) region of 380–780 nm is high for all films, 72–81%, which is important for TCO applications. Meanwhile, in the near infrared (NIR) region above 1200 nm, a difference in plasma absorption between the samples is observed. Since aluminum is acting as an acceptor doping in SnO${}_{2}$, a plasmon frequency shift towards the IR region is observed with increasing Al doping into the tin oxide films.

**Figure 7:**
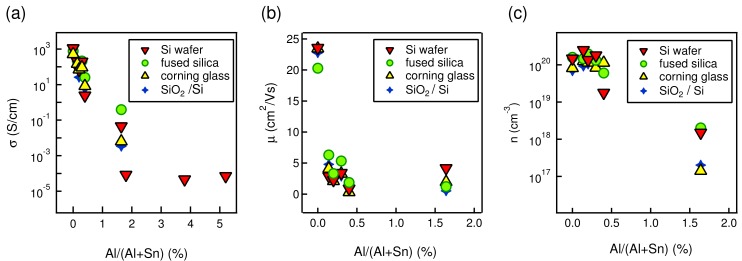
Conductivity (**a**), carrier mobility (**b**), and carrier concentration (**c**) of nominally undoped SnO${}_{2}$ and Al-doped SnO${}_{2}$ thin films with different compositions prepared on different substrates. Hall measurements were possible only for samples with composition up to SnO${}_{2}$:Al-1.64.

In the spectral range of relevant electromagnetic wavelengths for the applications in which TCOs are used (i.e., flat screens, solar cells, etc.), free electrons dominate the electrical and optical properties. These properties can be described in a first approximation by the Drude free electron theory [56]. This theory often accounts for the measurable properties of TCOs, such as transmittance and reflectance, and their relationship to extrinsically controllable parameters (such as carrier concentration) and intrinsically uncontrollable properties (such as crystal lattice and effective mass). The plasmon frequency shift towards higher wavelength observed upon Al doping is in a good agreement with the determined free carrier concentrations, see Figure 7c. Due to the fact that SnO${}_{2}$ is naturally n-type TCO, the transmittance drop in NIR region was ascribed to plasmonic absorption. Doping with aluminum favors the creation of holes as low valence Al${}^{3+}$ cations substitute Sn${}^{4+}$ ones. For this reason, Al${}^{3+}$ cations play the role of charge compensators and the transmittance in the INR region increases consequently, as shown by Figure 6b. The free carrier concentrations influence the resonant frequency of the plasma absorption according to the following relation within the framework of Drude theory [56]:

(3) $$\omega_{p}=\sqrt{\frac{ne^{2}}{m^{*}\varepsilon_{0}\varepsilon_{\infty }}},$$

where $\omega_{p}$ is plasma frequency, *n* is charge carrier density, *e* is elementary charge, $m^{*}$ is carrier effective mass, $\varepsilon_{0}$ is vacuum permittivity, and $\varepsilon_{\infty }$ is the value for the film dielectric constant at high frequencies.

For highly doped TCOs, the plasmon energies are up to 1 eV, which is in the near infrared region of the electromagnetic spectrum. Due to the high number of free electrons, the incident infrared radiation is not transmitted but is rather reflected; this is a key feature for application of differently doped tin oxide thin films as low-emissivity window coatings.

Conductivity of nominally undoped and the different Al-doped SnO${}_{2}$ thin films is provided in Figure 7a. Hall effect results of the same films are also displayed in Figure 7b for the carrier mobility and (c) for the carrier concentration. Due to the experimental limitations of the Hall effect setup used, no Hall effect could be measured for the doped films above SnO${}_{2}$:Al-1.64.

Our nominally undoped tin oxide films prepared on different substrates have a conductivity of ≈1.1 × 10${}^{3}$ S/cm. Surprisingly, this value is at least two orders of magnitude higher than the conductivity of undoped SnO${}_{2}$ films reported in literature. For magnetron sputtered films, the reported conductivity varies from 10${}^{-5}$ to 10${}^{0}$ S/cm depending on the different deposition conditions [21,22]. Similarly, SnO${}_{2}$ films prepared by PLD have conductivity values of ≤4.5 ×10${}^{1}$ S/cm [57], and that of sol-gel exhibit values ≤10${}^{0}$ S/cm [58]. The higher conductivity obtained for the thin films studied here (deposited by spray pyrolysis) is probably related to the tin precursor used, namely, SnCl${}_{4}$·5(H${}_{2}$O). Since chlorine has one less 2p orbital to fill than oxygen, substitution of O${}^{2-}$ ions by Cl${}^{-}$ ions leads to an increase of free electrons per SnO${}_{2}$ unit formula. For every chlorine substitution, a tin atom retains an extra 5 s electron which enters the conduction band of the lattice [39,59]. In addition, oxygen vacancies are deep donors in SnO${}_{2}$ [60,61], which can not be easily ionized and contribute to the electrical conduction. Eventually, we were not able to detect chlorine on our samples using different physicochemical analysis techniques.

In order to address the actual role of the incorporated chlorine atoms with respect to the electrical properties, Messad et al. [39] performed Rutherford back scattering (RBS) analyses on spray deposited SnO${}_{2}$ films from SnCl${}_{4}$ precursor to determine the bulk chloride concentration (*n*${}_{Cl^{-}}$) and compared it with the Hall carrier concentration (*n*) of the same films. Their results showed that for unintentionally doped SnO${}_{2}$ films deposited with precursor concentration of 0.1 M and substrate temperature between 400 and 550 ${}^{{}^{\circ}}$C, the chlorine ion concentration (*n*${}_{Cl^{-}}$) and Hall concentration (*n*) have the same values. Thus, they concluded that, within these experimental conditions, the carrier density can be even identified with chlorine content. Meanwhile, at a deposition temperature higher than 550 ${}^{{}^{\circ}}$C, the *n*${}_{Cl^{-}}$ decreased with increasing substrate temperature. This is due to the breakage of Sn-Cl bonds with increasing temperature, as it was evident with Auger electron spectroscopy (AES) [62] and Secondary ion mass spectroscopy (SIMS) [63] observations. Even though they [39] successfully demonstrated the incorporation of chlorine ions, under the same deposition conditions, they measured a conductivity of 6 × 10${}^{1}$ S/cm. This is in fact two orders of magnitude lower that the conductivity reported in the present article for nominally undoped films.

For Al doped films, the scenario is different. Since Al${}^{3+}$ ions substitute Sn${}^{4+}$, a hole is generated per SnO${}_{2}$ molecule and compensates an existing carrier electron. Thus, the conductivity of tin oxide films does not improve upon Al incorporation. The conductivity of SnO${}_{2}$:Al decreases consistently with increasing Al content due to the presence of compensating holes and, for the highest Al content (1.8, 3.8, and 5.2) films, the conductivity drops to ∼10${}^{-4}$ S/cm.

Hall effect measurements were possible only for the films with composition up to SnO${}_{2}$:Al-1.64, the last three films in the series being too resistive and thus impossible to be measured with our system. Hall mobility exhibits a pronounced reduction with small Al incorporation. It decreased from ≈23 cm${}^{2}$/Vs for undoped films to ≈5 cm${}^{2}$/Vs for SnO${}_{2}$:Al-0.15 samples. With further increasing Al concentration up to SnO${}_{2}$:Al-1.64, the mobility stayed in the following range: 1–5 cm${}^{2}$/Vs. This reduction in mobility could be attributed to the change in micro-structure of the films upon Al incorporation due to the grain size reduction for instance. Conversely, the carrier concentration was not affected in low Al content films with value of ≈ 1 × 10${}^{20}$ cm${}^{-3}$, being obtained for doping values up to 0.3. Then, it decreased for the SnO${}_{2}$:Al-0.4 film and finally dropped by three orders of magnitude ≈ 1 × 10${}^{17}$ cm${}^{-3}$ for SnO${}_{2}$:Al-1.64 samples.

The relation between the charge carrier concentration and mobility is rather complicated in polycrystalline semiconducting materials [64]. In general, these materials exhibit a vast amount of grain barriers depending on their mean grain size, which constitute crystallographically disturbed regions, leading to electronic defects in the band gap of semiconductors. These defects are charged by carriers from the grains. Charge balance causes a depletion zone on both sides of a grain barrier accompanied by an energetic barrier for the carriers [65]. The carriers therefore need to cross these energetic barriers and this is mostly a thermally activated process. Frischbier and coworkers [66] reported that grain boundaries may influence the carrier mobility even up to the carrier concentrations of 10${}^{21}$ cm${}^{-3}$. Usually, such a high carrier density is achieved by degenerately doping the semiconductor and ionized impurities should be the main scattering mechanism for the reduction of charge carrier mobility [65]. As we have seen in the morphological study, aluminum incorporation resulted in a heterogeneous nucleation of smaller grains which leads to the formation of more grain boundaries, which act as a potential barrier and reduce carrier mobility. Therefore, the observed reduction in mobility is reasonable. At the same time, Al doping favors the creation of holes as low valence Al${}^{3+}$ cations substitute Sn${}^{4+}$ ones. The created holes then compensate the existing carrier electrons and charge carrier density decreases. This is supported by the results of Hall effect measurements as carrier concentration decreased by up to three orders of magnitude only by incorporation of 1.64% of Al.

Some reports claim [34,35] that, for a high enough Al doping of tin oxide, the type inversion of conductivity could have been observed. This would mean having a tin oxide thin film with p-type conductivity. Mohagheghi et al. reported this transition can take place close to 8% of Al incorporated into their films [33]. Meanwhile, in our Hall effect results, we did not see such a type inversion behavior, probably because our SnO${}_{2}$:Al thin films did not have high enough Al content.

## 4. Conclusions

In summary, we have presented a comprehensive study describing the change in structural, optical, and electrical properties of tin oxide thin films prepared by ultrasonic spray pyrolysis upon Al incorporation. The doping was possible by Al${}^{3+}$ cations substituting Sn${}^{4+}$ sites in the SnO${}_{2}$ lattice, as deducted from TEM and XRD analyses.

SEM images showed a decreasing grain size with increasing Al content in the prepared tin oxide thin films. In addition, Al doping drastically decreased the number of planar twin defects present in tin oxide films. EPMA analyses revealed that only ∼1/3 of Al present in the precursor solution is actually transferred into the grown films.

Al doping also resulted in structural reordering of tin oxide by texture transition from (301) to (101), and then to (002) upon increasing Al content in the grown films. The optical transmittance of tin oxide was not changed in the visible region with an average transmittance of 72–81% being obtained. Meanwhile, a difference in plasmon absorption is observed in the near infrared region for all samples: the plasmon frequency shifted towards the IR region as a function of Al doping concentration.

Nominally undoped tin oxide films exhibit a conductivity of ≈1.1 × 10${}^{3}$ S/cm, which is at least two orders of magnitude higher than the conductivity reported for undoped tin oxide films. This higher conductivity stems from the SnCl${}_{4}$·5(H${}_{2}$O) precursor used during sample preparation, in which Cl${}^{-}$ ions act as an additional donor doping. Since aluminum is a low valence cation doping for SnO${}_{2}$, the conductivity of tin oxide films decreased with incorporation of Al.

These findings help to improve the understanding of the effect of Al doping on the properties of tin oxide thin films, including structural, optical, and electrical properties. In addition, the gained knowledge can be transferred to modify the properties of highly demanded florine doped tin oxide (FTO) thin films.

## Figures and Tables

**Table 1:** Al/(Al+Sn) ratio in solution and in the different Al-doped SnO${}_{2}$ thin films (from EPMA data): Film thickness from SEM cross-section and deposition time and rate of studied films. The deposition rates are given with uncertainty of ±0.5 nm/min.

Al/(Al+Sn) Solution	Al/(Al+Sn) Film	Film Thickness	Deposition Time	Deposition Rate
(%)	(%)	(nm)	(min)	(nm/min)
0	-	670	45	15
0.5	0.15	260	25	10.4
1	0.2	340	30	11.3
1.37	0.3	260	25	10.4
1.96	0.4	290	25	11.6
2.44	1.64 ± 0.08	200	30	6.7
4.76	1.8 ± 0.08	660	45	14.66
9.1	3.8 ± 0.2	800	45	17.7
13.04	5.2 ± 0.5	580	45	13

## Abbreviations

The following abbreviations are used in this manuscript:

SnO${}_{2}$:Al	Al-doped tin oxide
ITO	Sn-doped indium oxide
FTO	F-doped tin oxide
AZO	Al-doped zinc oxide
TCO	Transparent conducting oxide
XRD	X-ray diffraction
SEM	Scanning electron microscopy
EDS	Energy-dispersive spectroscopy
TEM	Transmission electron microscopy
EDX	Energy-dispersive X-ray spectroscopy
FTIR	Fourier-transform infrared Spectroscopy
ALD	Atomic layer deposition
CVD	Chemical vapor deposition
EPMA	Electron probe micro-analyzer
SIMS	Secondary ion mass spectroscopy
AES	Auger electron spectroscopy
RBS	Rutherford back scattering
